# Multi-Task NoisyViT for Enhanced Fruit and Vegetable Freshness Detection and Type Classification

**DOI:** 10.3390/s25195955

**Published:** 2025-09-24

**Authors:** Siavash Esfandiari Fard, Tonmoy Ghosh, Edward Sazonov

**Affiliations:** Department of Electrical and Computer Engineering, The University of Alabama, Tuscaloosa, AL 35401, USA; tghosh@crimson.ua.edu

**Keywords:** fruit freshness detection, fruit and vegetable freshness detection, vision transformer, multi-task learning, computer vision, sensor-based classification, personal healthcare

## Abstract

Freshness is a critical indicator of fruit and vegetable quality, directly affecting nutrition, taste, safety, and reducing waste across supply chains. Accurate detection is essential for quality control, supporting producers during harvesting and storage, and guiding consumers in purchasing decisions. Traditional manual assessment methods remain subjective, labor-intensive, and susceptible to inconsistencies, highlighting the need for automated, efficient, and scalable solutions, such as the use of imaging sensors and Artificial Intelligence (AI). In this study, the efficacy of the Noisy Vision Transformer (NoisyViT) model was evaluated for fruit and vegetable freshness detection from images. Across five publicly available datasets, the model achieved accuracies exceeding 97% (99.85%, 97.98%, 99.01%, 99.77%, and 98.96%). To enhance generalization, these five datasets were merged into a unified dataset encompassing 44 classes of 22 distinct fruit and vegetable types, named Freshness44. The NoisyViT architecture was further expanded into a multi-task configuration featuring two parallel classification heads: one for freshness detection (binary classification) and the other for fruit and vegetable type classification (22-class classification). The multi-task NoisyViT model, fine-tuned on the Freshness44 dataset, attained outstanding accuracies of 99.60% for freshness detection and 99.86% for type classification, surpassing the single-head NoisyViT model (99.59% accuracy), conventional machine learning and CNN-based state-of-the-art methodologies. In practical terms, such a system can be deployed across supply chains, retail settings, or consumer applications to enable real-time, automated monitoring of fruit and vegetable quality. Overall, the findings underscore the effectiveness of the proposed multi-task NoisyViT model combined with the Freshness44 dataset, presenting a robust and scalable solution for the assessment of fruit and vegetable freshness.

## 1. Introduction

Fruits and vegetables, rich in vitamins, dietary fibers, and essential nutrients, play a critical role in managing weight and promoting overall health [1]. Maintaining their freshness is crucial, as it directly influences their nutritional value, flavor, and reduces waste generation [2,3]. Moreover, precise freshness assessment significantly enhances logistical efficiency, particularly in storage and transportation [4]. However, traditional manual freshness evaluation methods, performed by trained inspectors for color and firmness [5], are often subjective, inefficient, and costly [3,6], where sorting sweet potatoes in packaging facilities accounts for 30–50% of total labor costs [7], underscoring a critical need for automated solutions to improve accuracy, speed, and cost-effectiveness. In [8], the net annual benefit for apple orchards was estimated at $13,500–$78,400 when machinery costs ranged from $100k to $160k for large-scale operations, while equipment priced below $30k was suggested to be cost-effective for typical fresh-apple growers [9]. To address this need, contemporary research increasingly emphasizes data-driven methodologies, particularly those leveraging image analysis and quantifiable features to classify freshness effectively.

Use of imaging sensors (such as RGB, multispectral, and depth cameras) in combination with computer vision methods presents a compelling alternative both for industrial and personal use. In a systematic review on the use of computer vision and artificial intelligence for fruit inspection and classification [10] RGB cameras emerged as the most common data acquisition hardware, being employed in 84% of the studies, underscoring the critical role of camera-based models in this field. In [11], tomato maturity assessment using a lightweight, improved YOLOv8n achieved 98.7% precision and 99.2% mAP. An automated apple detection and counting system was introduced in [12], where an improved YOLO model processed video frames reached 97.4% precision and 96.4% mAP. Automated mango harvesting was explored in [13], YOLOv8 and YOLOv9 were used to detect, count, and estimate fruit size in orchards. Fine-tuning YOLOv7 with an attention mechanism on apple images improved detection and counting performance [14]. Additionally, ref. [15] applied a fuzzy model utilizing nine handcrafted features from the Fruit-360 dataset, achieving a classification accuracy of 98.36%, and [16] employed an enhanced YOLOv8-based fruit detector with a dynamic Kalman filter featuring a variable forgetting factor for robust fruit tracking. RGB cameras are also becoming common in modern wearable sensors. For example, AIM-2, a passive wearable device combining an accelerometer and a camera, has been proposed to automatically detect food intake and capture images during eating episodes [17], and for food type recognition [18]. Similarly, ref. [19] demonstrated that combining accelerometer and camera data for eating detection achieved an F1-score of 80.77%. A real-time eating monitoring system using images captured by a camera was presented in [20].

Rapid advancements and widespread adoption of Machine Learning (ML) classifiers, particularly supervised algorithms for categorical prediction, have led to the successful implementation of various algorithms, such as XGBoost [21] and regression-based approaches [22], across multiple domains, including fruit and vegetable freshness classification [23]. Specifically, in [24], the XGBoost classifier achieved accuracies of 93.3% and 98.1% on two separate datasets, each comprising three fruit types. Moreover, ref. [25] employed Support Vector Machine (SVM), K-Nearest Neighbors (KNN), and Decision Trees to classify fruit freshness based on moisture content. Another study [26] reported approximately 77% accuracy using Decision Trees, KNN, SVM, and Random Forest classifiers for the freshness detection of apples, bananas, and oranges. Furthermore, in [27], SVM models evaluating gas concentrations (O_2_, CO_2_, C_2_H_4_) within containers demonstrated superior accuracy in freshness determination. Reinforcement learning has shown potential in addressing complex tasks [28], such as optimizing ripe strawberry harvesting while protecting unripe strawberries from damage [29].

Recent developments in neural networks, particularly Convolutional Neural Networks (CNNs) and deep learning models, have introduced robust analytical frameworks for various tasks, including anomaly detection [30], object detection [31], and semantic segmentation [32], Generative Adversarial Networks (GAN) [33], and classification tasks [34,35], such as medical diagnostics [36], fruit identification, and freshness detection [37]. Customized CNN architectures [38,39] and conventional architectures like DenseNet-201 [40] have delivered strong performance. For instance, ref. [41] utilized features extracted from GoogLeNet, DenseNet-201, and ResNeXt-101, combined with Principal Component Analysis (PCA) and an SVM classifier, and achieved an accuracy of 96.98% for vegetable and fruit freshness detection. Similarly, a dual-headed CNN designed for the simultaneous classification of fruit types and freshness was proposed in [6]. A CNN employing MobileNetV3 architecture and Inverted Residual Blocks successfully performed freshness classification [3]. Integration of CNN with Bidirectional Long Short-Term Memory (LSTM) further enhanced accuracy, achieving 97.76% across six different fruits and vegetables [1].

Following their introduction in 2017 [42], transformer-based models emerged as strong candidates for multimodal fusion [43], and have consistently outperformed CNNs across various domains, including time-series analysis [44,45,46], label cleaning [47], and image classification tasks [48], yielding superior results [49]. Specifically, transformers have shown remarkable performance in freshness detection. In [50], transformers notably exceeded CNN accuracy across two datasets. Moreover, ref. [4] demonstrated that vision transformers achieved an accuracy of 97.94%, surpassing CNN models such as ResNet152 and ConvNeXt, along with traditional ML techniques. Additionally, Swin Transformer and ViT effectively identified ripe and overripe apples and pears [51]. ViT and Swin Transformer achieved nearly 99% accuracy for apple and lettuce freshness detection [52], a transformer encoder reached 97% accuracy for papaya classification [53], and a hybrid attention transformer combined with YOLOv8 attained an mAP of 88.9% for ripeness classification across five fruit types [54]. Most existing methods focus on a limited number of fruit and vegetable classes, highlighting a significant research gap in developing a category-rich dataset for assessing freshness and enhancing classification accuracy.

This paper aims to enhance fruit and vegetable freshness detection accuracy and generalizability through an advanced multi-task Noisy Vision Transformer (NoisyViT) model. Recognizing the absence of a comprehensive dataset for freshness detection, five publicly available datasets were merged into a unified dataset comprising 22 distinct fruit and vegetable types, categorized as fresh or rotten, named Freshness44. Since manual freshness assessment is subjective, inconsistent, and inefficient, this work explicitly proposes a multi-task NoisyViT framework that simultaneously performs freshness detection (fresh vs. rotten) and fruit/vegetable type classification (22 classes). To effectively perform classification of freshness and item type, the NoisyViT architecture was expanded into a multi-task configuration with two dedicated heads: one for binary freshness classification, and the other for 22-class fruit or vegetable type classification. Both the single-task and multi-task NoisyViT models were initialized using pretrained ImageNet weights and subsequently fine-tuned on individual and merged datasets, respectively. The key contributions of this paper are (1) creation of the category-rich Freshness44 dataset for fruit and vegetable freshness and type classification, (2) extension of the NoisyViT model into a multi-head architecture for simultaneous freshness and type classification, and (3) comprehensive evaluation comparing the proposed approach against traditional ML techniques, CNN-based methods, and single-head transformer models, demonstrating superior performance of the multi-task NoisyViT.

The remainder of this paper is structured as follows: Section 2 details the methodology and datasets used; Section 3 presents fine-tuning results; Section 4 discusses the implications of these findings; Section 5 presents limitations of the work; and Section 6 offers concluding remarks.

## 2. Materials and Methods

### 2.1. Dataset

Image datasets related to fruits and vegetables typically fall into two main categories: classification by fruit or vegetable type, where each class represents images of a specific type, and freshness classification, in which each type is further divided into multiple freshness levels. Recent progress in image classification models has led to an increase in datasets dedicated specifically to fruit and vegetable freshness detection. These datasets are commonly created through self-collected imagery, web scraping, or a combination of both methods. However, existing datasets usually focus on a limited selection of fruits and vegetables, and, to our knowledge, no comprehensive dataset currently exists that covers an extensive variety of produce. Therefore, multiple fruit and vegetable freshness datasets were utilized in this study to facilitate a more thorough analysis. None of the datasets were pre-partitioned to training, validation, and test subsets; therefore, all datasets were divided into 60% for training, 20% for validation, and 20% for testing.

#### 2.1.1. Fresh and Stale Images of Fruits and Vegetables Dataset

This dataset includes images of six fruits and vegetables: apple, banana, bitter gourd, capsicum, orange, and tomato, each categorized into fresh and stale classes. The dataset was created using a self-collection approach, capturing daily images of the produce with a smartphone and supplemented by selected frames extracted from videos to increase the dataset volume, and were labeled based on visual inspection. The dataset consists of 14,682 images and is publicly accessible [1]. The characteristics of this dataset are summarized in Table 1.

#### 2.1.2. Fruits and Vegetables Dataset

This dataset contains images of five fruits (banana, apple, orange, mango, and strawberry) and five vegetables (potato, cucumber, carrot, tomato, and bell pepper). Each type is divided into fresh and rotten categories. Images were sourced from various online platforms, including Google Images, Bing Images, Kaggle, and Fruit360. The dataset comprises roughly 12,000 images, with approximately 600 per class, and it is publicly accessible (https://www.kaggle.com/datasets/muhriddinmuxiddinov/fruits-and-vegetables-dataset (accessed on 10 September 2022)) [55]. A summary of the dataset’s key characteristics is presented in Table 1.

#### 2.1.3. Fresh and Rotten Fruits Dataset for Machine-Based Evaluation of Fruit Quality

This dataset encompasses eight fruit types: apples, bananas, oranges, grapes, guavas, jujubes, pomegranates, and strawberries. Each fruit type is classified into fresh and rotten categories. Images were captured with a Nikon D5600 single-lens reflex digital camera, featuring a 23.5 × 15.6 mm CMOS sensor and a resolution of 24.2 million pixels. Initially, 3200 original images (200 per class) were obtained. The dataset was expanded with 12,335 augmented images. All images were annotated with guidance from an agricultural expert [6,56]. Full details of these dataset characteristics can be found in Table 1.

#### 2.1.4. FruitNet

The FruitNet dataset comprises images of six fruits: apple, banana, guava, lime, orange, and pomegranate, classified into three quality categories: good quality (fresh), bad quality (rotten), and mixed quality (containing both fresh and rotten samples in a single image). The dataset comprises a total of 19,526 images. Images were captured under various backgrounds and lighting conditions in both indoor and outdoor settings, utilizing high-resolution rear cameras from three different mobile devices: iPhone 6 (Apple, Cupertino, CA, USA), ZUK Z2 Plus (ZUK Mobile, Beijing, China), and Realme 5 Pro (Realme, Shenzhen, China) [57]. Table 1 provides a summary of FruitNet’s characteristics.

#### 2.1.5. FruitQ

This dataset was constructed by manually extracting frames from YouTube videos (Google, San Bruno, CA, USA) featuring 11 fruit types: banana, cucumber, grape, kaki, papaya, peach, avocado, pepper, strawberry, tomato, and watermelon. Each image was manually annotated based on freshness quality into three categories: Fresh, Mild, and Rotten. The dataset contains a total of 9421 images, with varying numbers of images across the classes [2]. The main properties of FruitQ are summarized in Table 1.

#### 2.1.6. Freshness44 Dataset

Due to the lack of a comprehensive dataset focused on fruit and vegetable freshness detection, the aforementioned datasets were merged in the final stage of this study. This merging process allowed for the inclusion of a broader range of fruit and vegetable types for classification. Furthermore, integrating multiple datasets, some of which contain overlapping categories, enhanced the overall diversity and richness of the Freshness44 dataset. This enhancement is due to the variations in illumination, perspectives, backgrounds, and image resolutions present within the individual datasets [58].

**Figure 1:**
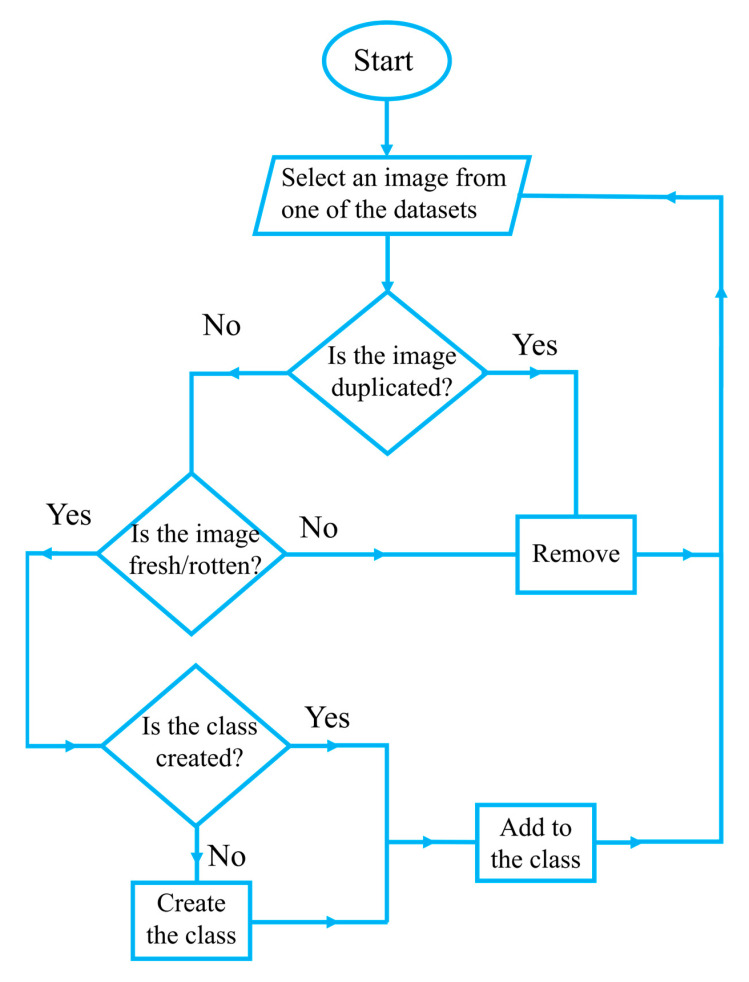
Flowchart of the Freshness44 dataset creation process.

To construct the Freshness44 dataset, distinct fruit and vegetable categories were identified, leading to 22 unique types. All images from various datasets corresponding to each class were included, yielding a total of 53,616 images, with a mixed resolution, ranging from 144 × 122 pixels to 8000 × 6000 pixels. Among the five datasets, FruitNet comprises three classes: fresh, rotten, and mixed quality (images containing both fresh and rotten items). Since the proposed approach focuses exclusively on image classification rather than object detection, the mixed-quality images were omitted from the Freshness44 dataset. Similarly, the FruitQ dataset includes three freshness levels: fresh, mild, and rotten. Given that our objective involves binary freshness classification (fresh versus rotten), images labeled as mild were also excluded from the Freshness44 dataset. The flowchart of creating this dataset is shown in Figure 1.

The different fruit/vegetable items and the number of images in each category are presented in Table 2.

**Figure 2:**
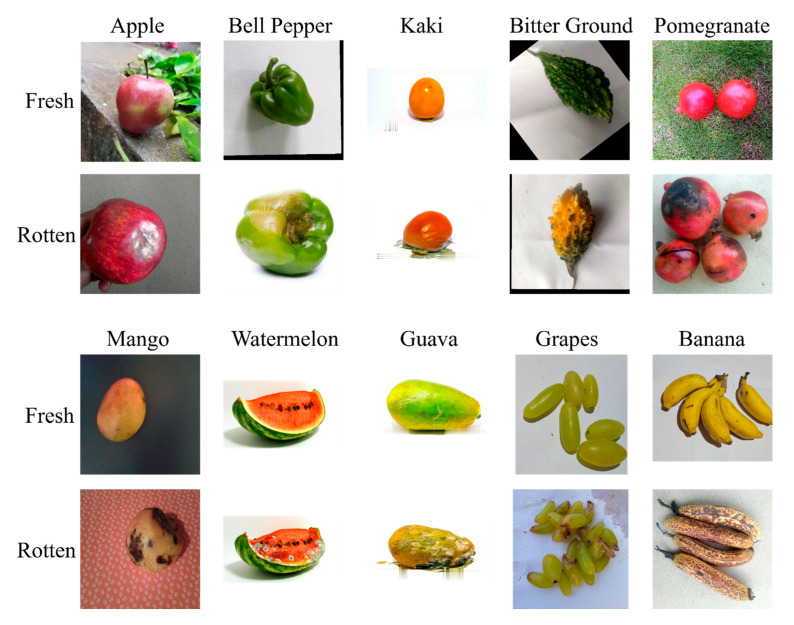
Selection of fruits and vegetables from various classes in the Freshness44 dataset.

To remove redundancy, MD5 hashing identified and eliminated duplicate images [50]. For consistency, all images were converted to JPEG format. A selection of fruit and vegetable samples is shown in Figure 2.

### 2.2. Data Preprocessing and Augmentation

To enhance classification performance using Vision Transformers (ViTs) and to increase the number of images for classes with fewer samples, the RandAugment method was applied to the training images, following the approach described in [59,60]. Two random sequential augmentations with a magnitude of nine were applied using ‘Nearest’ interpolation. Subsequently, random cropping, horizontal flipping (with a probability of 0.5), and normalization by subtracting mean values and dividing by standard deviation were applied. Validation and test images underwent resizing to match the ViT input dimension of 224 × 224 (or 384 × 384) pixels and normalization without additional augmentation. To maintain the original aspect ratio and prevent distortion when resizing rectangular images, each image’s shorter side is scaled first to the input dimension of the Vision Transformer. Then, a central square region that matches the model’s required resolution is cropped.

### 2.3. Multi-Task Noisy Vision Transformer (NoisyViT) Framework

The Noisy Vision Transformer (NoisyViT) is introduced as a versatile framework designed for single-object classification tasks, capable of accurately identifying individual items within an image [61]. Unlike traditional approaches, noise injection in this model is strategically employed not merely as regularization but to simplify the learning task by effectively reducing its complexity. Specifically, carefully selected noise has been shown to enhance the performance of deep learning models under certain conditions. To quantify the complexity of a classification task, Shannon entropy for a discrete variable *x* is utilized:

(1) $$H\left(x\right)=\;-\sum_{x}p\left(x\right)\log\left(p\left(x\right)\right)$$

When noise *ϵ* is injected into a classification task *T*, the resulting change in task entropy is defined as:

(2) $$∆S\left(T,\epsilon \right)=H\left(T\right)-H\left(T|\epsilon \right)$$

By evaluating how different perturbations impact the task entropy, the noise can be categorized as either “positive” (reducing task entropy), $∆S\left(T,\epsilon \right)>0$ or “harmful” (increasing task entropy, $∆S\left(T,\epsilon \right)\leq 0$). In practice, noise is injected into a randomly selected layer, and the resulting entropy change is subsequently analyzed. Empirical analyses indicated that Gaussian or Salt and Pepper noise typically increased task entropy, thereby complicating the learning process. In contrast, linear transform noise, achieved by applying a linear transformation matrix *Q* to input features *X* (denoted as *QX*), resulted in the entropy change defined as

(3) $$∆S\left(T,QZ\right)=-\log\left|I+Q\right|$$

where minimizing $\left|I+Q\right|$ effectively reduces entropy, simplifying the task. Thus, the application of linear transform noise becomes an optimization problem, aiming to maximize entropy reduction. The optimal linear transform $Q_{optimal}\in R^{k\times k}$, where *k* is the number of data samples, is obtained by

(4) $$Q_{optimal}=diag\left(\frac{1}{k+1}-1,\;\ldots ,\frac{1}{k+1}-1\right)+\frac{1}{k+1}1_{k\times k}$$

Once identified, the optimal layer is fixed for both training and testing phases, achieving the entropy change upper bound:

(5) $$∆S\left(T,Q_{optimal}X\right)=\left(k-1\right)\log\left(k+1\right)$$

Notably, the upper bound of entropy change was shown to depend on the dataset size, indicating that the benefits of positive noise are more pronounced in larger datasets. Due to the inherent regularization provided by the remaining layers in the model, introducing noise into a single layer was found to be sufficient [61].

In this work, positive noise injection was specifically implemented using the optimal linear transform *Q_optimal_* within the latent space of the transformer. Perturbations were applied to the final layers, reducing task entropy and thereby improving generalization. This design was particularly effective for the Freshness44 dataset, as its larger sample size allowed the injected positive noise to approach the theoretical upper bound of entropy reduction, leading to improved robustness in both freshness detection and type classification tasks. The mechanism can be intuitively understood as a subtle circular shift, where samples (images) are nudged slightly toward their neighbors, reducing within-class variance and making label-predictive features more distinct, while maintaining between-class separation.

Unlike traditional regularization methods such as dropout or weight decay, which lack an explicit optimization formulation, positive noise injection through linear transforms is grounded in entropy minimization. While conventional regularization primarily mitigates overfitting, positive noise directly decreases task complexity, improving both convergence and final accuracy. Furthermore, as dataset size increases, the benefits of entropy reduction become more pronounced, making this approach particularly well-suited to large-scale datasets like Freshness44.

This study employed the base Vision Transformer (ViT) model, comprising 12 layers, a patch size 16, and input image resolution of 224 × 224 pixels. The model was initialized using pre-trained weights from ImageNet-1K and subsequently fine-tuned on the fruit and vegetable datasets for 30 epochs. Training utilized a learning rate of 1 × 10^−6^, the AdamW optimizer, cosine learning rate scheduler, and label-smoothing cross-entropy as the loss function.

**Figure 3:**
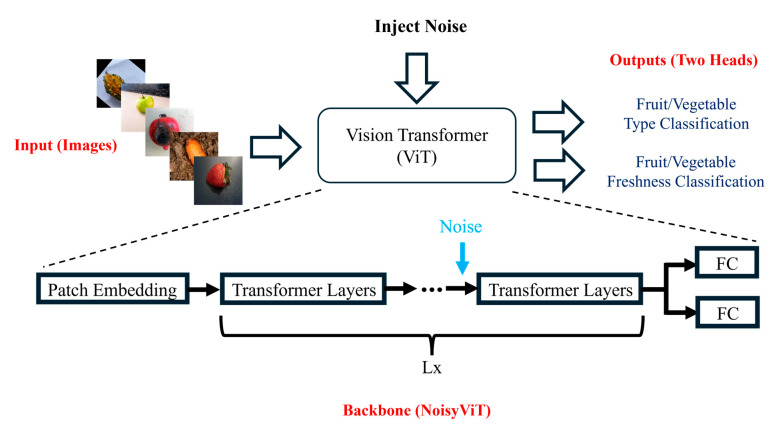
Multi-Task NoisyViT Architecture.

To further enhance the capabilities of NoisyViT, this study introduced a multi-task learning variant, shown in Figure 3. Multi-task learning leverages related task information to improve model generalization by employing a shared feature extractor with distinct output heads to handle interrelated predictions [62]. Given the goal of simultaneously predicting freshness detection and type classification for fruits and vegetables, the original NoisyViT model was adapted into a multi-task architecture. Specifically, two parallel heads were designed: a binary classification head for freshness detection and a 22-class classification head for identifying the fruit or vegetable type. Implementation involved loading the pretrained NoisyViT model, removing its original classification head to retain only the feature extraction layers (backbone), and then appending two new classification heads. During training, the total loss was computed as the sum of the individual losses from both heads. The backbone of the multi-task NoisyViT model was initialized with pretrained weights from ImageNet. Given that the additional heads were initialized randomly, the backbone parameters were initially frozen during the first three epochs, allowing only the weights and biases of the newly added heads to be trained at a learning rate of 0.001. After completing these initial three epochs, the backbone parameters were unfrozen, and the entire model underwent fine-tuning for 30 epochs using the established hyperparameters consistent with the single-task NoisyViT framework.

Model training and evaluation were conducted on a system equipped with a 9th Gen Intel^®^ Core™ i9-9900K CPU, 32 GB RAM, and an NVIDIA RTX 2080 Ti GPU. Following the completion of training, the model that achieved the highest validation accuracy was utilized for inference on the test set. Model performance was evaluated based on accuracy metrics. Additionally, the results were compared with those of state-of-the-art models to assess the relative effectiveness of the proposed approach.

The pretrained NoisyViT model was fine-tuned separately on each dataset using the respective training samples. Throughout training, model performance was monitored at each epoch by computing the accuracy on the corresponding validation set. The model that achieved the highest accuracy on the validation set was selected as the final model for evaluation on the test set. To mitigate the impact of data splitting on the classification outcomes of the single-head model, all datasets were randomly partitioned into training, validation, and test sets across 10 independent runs. The average accuracy from these 10 runs was then reported as the model’s final performance. The multi-task NoisyViT model, however, was exclusively fine-tuned on the Freshness44 dataset.

### 2.4. Performance Metrics and Evaluation Protocol

Model performance was assessed using standard classification metrics on the test set. To ensure comparability with previous studies, accuracy was calculated based on the total number of samples, defined as

(6) $$Accuracy=\frac{TP+TN}{TP+TN+FP+FN}$$

where *TP*, *TN*, *FP*, and *FN* represent the entries of the confusion matrix. To account for potential class imbalance across fruit and vegetable categories, precision was computed for each class, given by

(7) $$Precision=\frac{TP}{TP+FP}$$

which measures the proportion of correctly predicted positive samples out of all predicted positives. Similarly, recall was calculated as

(8) $$Recall=\frac{TP}{TP+FN}$$

indicating the proportion of correctly predicted positive samples out of all actual positives. Finally, the F1-score was derived as the harmonic mean of precision and recall, defined as

(9) $$F1-score=2\times \frac{Precision\times Recall}{Precision+Recall}$$

## 3. Results

During training, the performance of each classification head, freshness, and type was evaluated individually at every epoch using accuracy. The final model was selected based on the highest average accuracy across both heads on the validation set and subsequently used for test set evaluation.

### 3.1. Fresh and Stale Images of Fruits and Vegetables Dataset

For the Fresh and Stale Images of Fruits and Vegetables Dataset, fine-tuning the proposed NoisyViT model resulted in an accuracy of 99.85% ± 0.081% (mean ± SD) on the test set, outperforming all previously reported models trained on this dataset. As shown in Table 3, conventional CNN architectures such as VGG16 and GoogleNet, along with the customized CNN model described in [1], a CNN combined with bidirectional LSTM, and Vision Transformer-based models achieved comparatively lower accuracy scores than the NoisyViT. NoisyViT improved accuracy by at least 1.5% compared to the state-of-the-art methods.

### 3.2. Fruits and Vegetables Dataset

Using the Fruits and Vegetables Dataset, the NoisyViT was fine-tuned to classify images from 10 different fruit and vegetable types into fresh and rotten categories. The model achieved an accuracy of 99.01% ± 0.292% on the test set, outperforming previously reported methods. Specifically, in [41], an approach using deep features extracted from three pre-trained deep learning models—GoogLeNet, DenseNet-201, and ResNeXt-101—combined with PCA-based dimensionality reduction, attained an accuracy of 96.98%. In comparison, the customized CNN model proposed in [39] achieved an accuracy of 98.20%.

### 3.3. Fresh and Rotten Fruits Dataset for Machine-Based Evaluation of Fruit Quality

Fine-tuning the NoisyViT model on the Fresh and Rotten Fruits Dataset for Machine-Based Evaluation of Fruit Quality resulted in an accuracy of 98.96% ± 0.695% on the test set, outperforming prior models developed for this dataset. Table 3 shows an approximate 5.5% improvement in accuracy over the models proposed in [6], which utilized a multi-task convolutional neural network, and [24], which employed an XGBoost classifier.

### 3.4. FruitNet

The performance of the trained NoisyViT model on the FruitNet dataset, along with a comparison against alternative models, is summarized in Table 3. As demonstrated, NoisyViT achieved superior performance, attaining an accuracy of 99.77% ± 0.070% on the test set, thereby outperforming previously proposed conventional and customized CNN-based models.

### 3.5. FruitQ

On the FruitQ dataset, which includes 16 types of fruits categorized into three quality classes, fresh, mild, and rotten, the NoisyViT model achieved an accuracy of 97.98% ± 0.623% on the test set.

### 3.6. Freshness44

In the final evaluation phase, the NoisyViT model was trained on the Freshness44 dataset containing 22 distinct fruit and vegetable types, each labeled as either fresh or rotten. The evaluation involved two distinct configurations: a single-head model with 44 combined classes and a multi-task architecture featuring two heads, one dedicated to classifying fruit and vegetable types, and another specifically for freshness detection, summarized in Table 4. Initially, a standard Vision Transformer (ViT) model without noise injection was trained on the Freshness44 dataset, followed by training the NoisyViT model, which included linear transform noise injected into its final layer. Lastly, the multi-task NoisyViT was trained using the same dataset.

**Figure 4:**
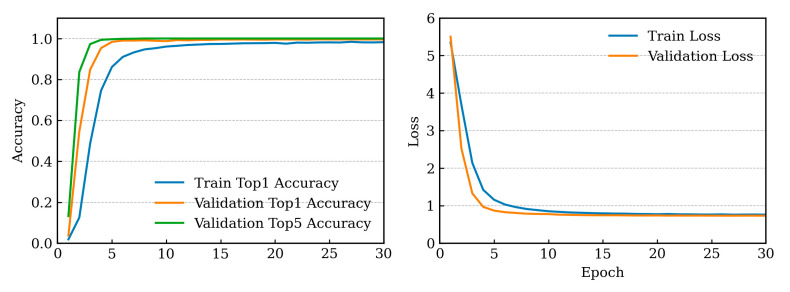
Training and validation results of the NoisyViT model.

As depicted in Figure 4, which illustrates training and validation loss curves over 30 epochs for the single-head NoisyViT model with 44 classes, both losses exhibited a steady decline during the first 15 epochs before stabilizing. A similar trend was also observed in the curves representing training accuracy.

**Figure 5:**
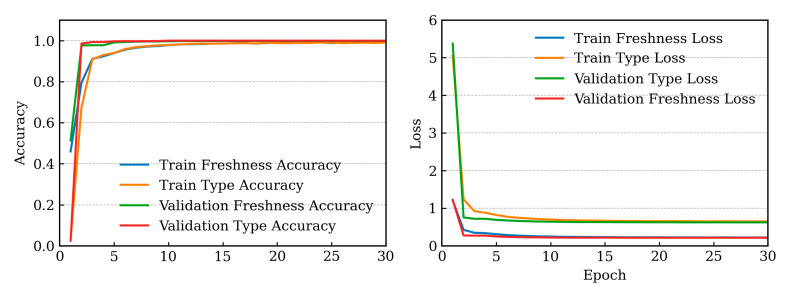
Training and validation results of the multi-task NoisyViT model.

For the multi-task configuration, the training and validation losses of both the freshness detection and type classification heads rapidly decreased within the initial five epochs, indicating rapid convergence, as illustrated in Figure 5. The standard ViT model achieved an accuracy of 99.32%. However, the NoisyViT model outperformed the standard ViT, achieving accuracies of 99.59% and 99.75% for image resolutions of 224 × 224 and 384×384, respectively. Moreover, the multi-task NoisyViT model achieved outstanding performance, reaching an accuracy of 99.60% and 99.65% for freshness detection and 99.86% and 99.84% for type classification for 224 × 224 and 384 × 384 image resolutions, respectively. When compared with the multi-task CNN in [6] at an image resolution of 224 × 224, the proposed multi-task NoisyViT outperformed CNN model, achieving 99.60% vs. 93.24% in freshness detection and 99.86% vs. 88.86% in type classification, while being trained on 44 classes from the Freshness44 dataset compared to 16 classes in the CNN model.

The confusion matrix for type classification using the proposed NoisyViT model with an input resolution of 224 × 224 on the test dataset is presented in Table 5. The class indices in the matrix correspond to the fruit and vegetable categories listed in Table 2. Based on this matrix, the average class-wise precision, recall, and F1-score are 99.69%, 99.79%, and 99.74%, respectively.

Similarly, the confusion matrix for freshness detection with the proposed NoisyViT model at an input resolution of 224 × 224 on the test dataset is shown in Table 6. From this matrix, the average class-wise precision, recall, and F1-score are 99.59%, 99.60%, and 99.60%, respectively.

## 4. Discussion

The creation of the merged dataset not only increased the number of fruit and vegetable classes and associated images per class but also enhanced model generalization by incorporating images taken under diverse lighting conditions, viewing angles, and background environments from multiple sources. In comparison, the Freshness44 dataset, featuring 22 distinct fruit and vegetable types, surpasses the previously mentioned datasets containing only 6, 5, 8, 6, and 11 classes, respectively. Furthermore, the Freshness44 dataset contains a total of 53,616 images, significantly exceeding the previously reported maximum of 19,526 images, thus making it highly suitable for data-driven methods. Practically, the trained multi-task NoisyViT model could be deployed on server-based infrastructure, facilitating real-time image capture and remote classification through internet connectivity. This deployment could substantially enhance efficiency and reliability in freshness evaluation processes.

As demonstrated in the Results section, various data-driven methodologies have been explored to enhance the accuracy of freshness detection in fruits and vegetables. Traditional machine learning techniques, such as XGBoost in Table 3, have achieved considerable accuracy levels, reaching up to 93.33%. Additionally, Convolutional Neural Networks (CNNs) have significantly advanced image classification performance across numerous applications, including datasets designed explicitly for freshness detection. Nevertheless, vision transformer models generally outperform traditional machine learning and CNN-based approaches due to their sophisticated attention mechanisms, enabling more effective feature representation. By using patch embeddings and positional encodings, ViTs treat the entire image as a sequence, allowing them to capture structural relationships across distant regions. Furthermore, multi-head self-attention flexibly combines information from different patches, enabling the model to focus on discriminative image regions while suppressing irrelevant background features [50].

The Noisy Vision Transformer (NoisyViT) utilized in this study specifically incorporates positive noise injection to simplify learning tasks by effectively reducing entropy. This strategy enhances model performance, positioning NoisyViT as superior to previously employed models in freshness detection tasks. Furthermore, the multi-task NoisyViT architecture introduced here allows the simultaneous learning of multiple related tasks, resulting in enhanced generalization and improved overall classification accuracy. An additional notable advantage of the NoisyViT model is its inherent regularization capability, implying that further augmentation of the dataset with diverse images could potentially yield additional improvements in classification performance. When fine-tuned on the Freshness44 dataset comprising 22 fruit and vegetable types labeled as fresh or rotten, the proposed single-head NoisyViT model achieved a remarkable accuracy of 99.59%. The multi-task NoisyViT model, equipped with two distinct heads dedicated to freshness detection and type classification, further improved these results, attaining accuracies of 99.60% and 99.86% for freshness detection and type classification, respectively. These outstanding performance metrics underscore the suitability of the multi-task architecture as a robust and reliable solution for real-time freshness assessment of fruits and vegetables. Additionally, the flexibility inherent to the multi-task approach allows the integration of further task-specific heads, such as ripeness detection or healthy versus unhealthy classification, thereby significantly broadening the model’s applicability across various food industry scenarios. NoisyViT may improve traditional classification pipelines by providing a principled, noise-based augmentation and enhancing robustness to variations in lighting, viewpoint, and acquisition device, advantages that are supported by training on Freshness44, a category-rich dataset compiled from multiple sources. Its modular multi-head design enables easy extension (for example, adding a ripeness-detection head), and the multi-task setup consolidates related outputs (freshness and type) into a single model, reducing inference calls and maintenance overhead. A promising real-world application of this framework lies in automated packaging lines, where conveyor-mounted cameras could capture produce images in real time, allowing the system to simultaneously determine freshness and type and enabling the immediate removal of spoiled items before packaging. Altogether, the proposed model may support a range of practical deployment pathways across consumer and industrial settings.

## 5. Limitations

Despite the high accuracy demonstrated by the multi-task NoisyViT model, several limitations remain. Firstly, the current model is constrained to classifying freshness in images containing either single or multiple instances of a single type of fruit or vegetable, provided all instances share the same freshness status (either fresh or rotten). Thus, images containing a combination of fresh and rotten fruits or vegetables, or multiple distinct fruit and vegetable items, present a challenge that the model cannot address effectively. Additionally, the current model does not support object localization, making it incapable of identifying the exact spatial position of fruits or vegetables within images. Unlike classical machine learning approaches with hand-crafted features, the NoisyViT framework relies entirely on automatically learned representations, reducing control over the features used. As with most deep learning architectures, interpretability remains limited, making it difficult to fully explain the rationale behind individual predictions. Future work could incorporate interpretability techniques, such as attention maps, to highlight influential image regions and improve model transparency. Moreover, the computational demands of the model introduce latency on edge devices, suggesting that GPU acceleration is required to ensure smoother real-time performance.

Another significant limitation relates to the dataset’s comprehensiveness. Although substantial efforts have been made to merge various sources, the availability of diverse and comprehensive datasets covering an extensive range of fruits and vegetables remains limited. This constraint affects the model’s generalizability and adaptability to additional fruit and vegetable types not included in the training dataset. Furthermore, real-world deployment scenarios, characterized by varied environmental conditions and occlusions, pose additional challenges that were not extensively explored in this study, underscoring the need for further research to ensure model robustness in practical, real-world applications. In addition, Freshness44 defines freshness as a binary label (fresh vs. rotten). In many practical settings, however, produce may fall into an intermediate, still-edible state that is neither fully fresh nor truly rotten. Because such borderline cases are not represented in the training data, the model’s performance on these intermediate-quality samples remains uncertain and warrants dedicated investigation in future work.

## 6. Conclusions

This study introduced and evaluated a multi-task variant of the Noisy Vision Transformer (NoisyViT) designed to automate fruit and vegetable freshness detection from imaging sensors. By integrating positive noise within the transformer architecture, NoisyViT effectively reduces learning complexity and significantly enhances classification accuracy. Initially, NoisyViT was fine-tuned across five distinct datasets for freshness detection, consistently outperforming previously established methods. To further improve generalization and establish a more diverse and category-rich benchmark, these five datasets were combined into a unified dataset comprising 22 fruit and vegetable types, each categorized as either fresh or rotten, named Freshness44.

The primary contribution of this research is the development and implementation of a multi-task NoisyViT architecture, employing a shared feature extractor complemented by two dedicated classification heads, one specifically for freshness detection and the other for identifying fruit or vegetable types. When trained on the Freshness44 dataset, the multi-task model achieved remarkable accuracies of 99.60% for freshness detection and 99.86% for type classification, clearly demonstrating superior performance compared to single-task configurations and previous models. Furthermore, this multi-task architecture offers considerable flexibility, enabling easy integration of additional tasks such as ripeness evaluation or healthy versus unhealthy classification, thus expanding its applicability across various agricultural and food quality assessment scenarios.

The exceptional accuracy, rapid inference capability, and suitability for real-time applications underscore the proposed multi-task NoisyViT model as a robust and scalable solution for intelligent freshness detection systems. While Freshness44 can be further expanded by incorporating additional fruit and vegetable categories, it already provides a diverse, category-rich, and standardized benchmark dataset for future studies. The dataset is publicly available and can be readily downloaded by researchers to design and evaluate models trained for real-world fruit and vegetable quality monitoring applications. Likewise, the multi-task NoisyViT establishes a strong baseline model whose high accuracy and multi-task structure can guide comparisons, adaptations, and extensions (by adding more heads) in subsequent research. Moreover, the multi-task framework is not limited to food classification tasks; it can also be applied to other domains requiring multiple prediction heads, such as medical imaging where one head may classify disease type while another estimates severity level. Its capacity for swift and reliable classification makes it particularly suitable for practical implementations, including server-based deployments that remotely process images captured and transmitted in real-time via internet connectivity for immediate freshness evaluation.

## Figures and Tables

**Table 1:** Dataset Characteristics: Classes, Image Counts, and Resolutions.

Dataset	Number of Categories (Fruit/Vegetable)	Number of Images	Image Resolution
Fresh and Stale Images of Fruits and Vegetables	6	14,682	Mixed from 144 × 122 to 862 × 386
Fruits and Vegetables	10	12,000	Mixed from 80 × 100 to 6183 × 4126
Fresh and Rotten Fruits	8	3200 with 12,335 Augmented Images	Mixed from 251 × 577 to 4160 × 3120
FruitNet	6	19,526	Mixed from 144 × 256 to 8000 × 6000
FruitQ	11	9421	Mix of 400 × 400 and 1280 × 720

**Table 2:** Number of images for each class in the Freshness44 dataset.

Fruit/Vegetable Type	Freshness	Number of Images	Fruit/Vegetable Type	Freshness	Number of Images
Apple	Fresh	3468	Lime	Fresh	1094
	Rotten	4263		Rotten	1085
Banana	Fresh	3513	Mango	Fresh	389
	Rotten	3605		Rotten	593
Bell Pepper	Fresh	1634	Orange	Fresh	3164
	Rotten	2108		Rotten	3443
Bitter Gourd	Fresh	327	Papaya	Fresh	130
	Rotten	357		Rotten	413
Carrot	Fresh	605	Peach	Fresh	425
	Rotten	507		Rotten	584
Cucumber	Fresh	833	Pear	Fresh	504
	Rotten	692		Rotten	100
Grape	Fresh	227	Pomegranate	Fresh	6140
	Rotten	288		Rotten	1387
Grapes	Fresh	200	Potato	Fresh	602
	Rotten	200		Rotten	562
Guava	Fresh	1352	Strawberry	Fresh	803
	Rotten	1329		Rotten	795
Jujube	Fresh	200	Tomato	Fresh	1905
	Rotten	200		Rotten	2504
Kaki	Fresh	545	Watermelon	Fresh	51
	Rotten	340		Rotten	150

**Table 3:** Classification Accuracy Comparison of NoisyViT and Prior Models on Different Datasets.

Dataset	Model	Accuracy
Fresh and Stale Images of Fruits and Vegetables Dataset	VGG 16 [1]	82.2%
	GoogLeNet [1]	94.62%
	CNN_BiLSTM [1]	97.76%
	Proposed Custom CNN model in [50]	97.65%
	Proposed ViT model in [50]	98.34%
	**NoisyViT (224 × 224)**	**99.85%**
Fruits and Vegetables Dataset	Combined Deep Features and PCA [41]	96.98%
	Customized CNN model [39]	98.20%
	**NoisyViT (224 × 224)**	**99.01%**
Fresh and Rotten Fruits Dataset for Machine-Based Evaluation of Fruit Quality	XGBoost [24]	93.33%
	Multi-Task CNN [6]	93.24%
	**NoisyViT (224 × 224)**	**98.96%**
FruitNet	ResNEt 152 [40]	97.86%
	VGG 16 [40]	98.6%
	Xception [40]	98.98%
	DenseNet201 [40]	99.26%
	XAI-FruitNet [38]	97.01%
	**NoisyViT (224 × 224)**	**99.77%**
FruitQ	**NoisyViT (224 × 224)**	**97.98%**

**Table 4:** Classification Accuracy of NoisyViT and Multi-Task NoisyViT Models on Freshness44.

Model	Image Resolution	Accuracy	Freshness Accuracy	Type Accuracy
Ordinary ViT	224 × 224	99.32%	-	-
Noisy ViT	224 × 224	99.59%	-	-
Noisy ViT	384 × 384	99.75%	-	-
Multi-Task Noisy ViT	224 × 224	99.73%	99.60%	99.86%
Multi-Task Noisy ViT	384 × 384	99.75%	99.65%	99.84%

**Table 5:** Type Classification Confusion Matrix for Multi-Task NoisyViT (224 × 224) on the Freshness44 Dataset.

Class	01	02	03	04	05	06	07	08	09	10	11	12	13	14	15	16	17	18	19	20	21	22
01	1545	1	0	0	0	0	0	0	0	0	0	0	0	0	0	0	0	0	0	0	1	0
02	0	1424	0	0	0	0	0	0	0	0	0	0	0	0	0	0	0	0	0	0	0	0
03	0	1	746	0	0	2	0	0	0	0	0	0	0	0	0	0	0	0	1	0	0	0
04	0	0	0	138	0	0	0	0	0	0	0	0	0	0	0	0	0	0	0	0	0	0
05	0	0	0	0	223	0	0	0	0	0	0	0	0	0	0	0	0	0	0	0	0	0
06	0	0	0	0	0	305	0	0	0	0	0	0	0	0	0	0	0	0	1	0	0	0
07	0	0	0	0	0	0	104	0	0	0	0	0	0	0	0	0	0	0	0	0	0	0
08	0	0	0	0	0	0	0	80	0	0	0	0	0	0	0	0	0	0	0	0	0	0
09	0	0	0	0	0	0	0	0	537	0	0	0	0	0	0	0	0	0	0	0	0	0
10	0	0	0	0	0	0	0	0	0	80	0	0	0	0	0	0	0	0	0	0	0	0
11	0	0	0	0	0	0	0	0	0	0	177	0	0	0	0	0	0	0	0	0	0	0
12	0	0	0	0	0	0	0	0	0	0	0	436	0	0	0	0	0	0	0	0	0	0
13	0	1	1	0	0	0	0	0	0	0	0	0	193	0	0	0	0	0	2	0	0	0
14	0	0	0	0	0	0	0	0	0	2	0	0	0	1320	0	0	0	0	0	0	0	0
15	0	0	0	0	0	0	0	0	0	0	0	0	0	0	109	0	0	0	0	0	0	0
16	0	0	0	0	0	0	0	0	0	0	0	0	0	0	0	202	0	0	0	0	0	0
17	0	0	0	0	0	0	0	0	0	0	0	0	0	0	0	0	121	0	0	0	0	0
18	0	0	0	0	0	0	0	0	0	0	0	0	0	0	0	0	0	1506	0	0	0	0
19	0	0	0	0	0	1	0	0	0	0	0	0	2	0	0	0	0	0	231	0	0	0
20	0	0	0	0	0	0	0	0	0	0	0	0	0	0	0	0	0	0	0	320	0	0
21	1	0	1	0	0	0	0	0	0	0	0	0	0	0	0	0	0	0	0	0	880	0
22	0	0	0	0	0	0	0	0	0	0	0	0	0	0	0	0	0	0	0	0	0	41

**Table 6:** Freshness Classification Confusion Matrix for Multi-Task NoisyViT (224 × 224) on the Freshness44 Dataset.

Class	Fresh	Rotten
Fresh	5611	17
Rotten	26	5082

## Data Availability

Dataset is publicly available (https://www.kaggle.com/datasets/siavash93/freshness44 (accessed on 10 August 2025)).

## Abbreviations

The following abbreviations are used in this manuscript:

AI	Artificial Intelligence
ViT	Vision Transformer
CNN	Convolutional Neural Network
ML	Machine Learning
LSTM	Long Short-Term Memory
SVM	Support Vector Machine
KNN	K-Nearest Neighbors
GAN	Generative Adversarial Network
